# Bryonolic Acid Blocks Cancer Cell Clonogenicity and Invasiveness through the Inhibition of Fatty Acid: Cholesteryl Ester Formation

**DOI:** 10.3390/biomedicines6010021

**Published:** 2018-02-12

**Authors:** Farid Khallouki, Robert Wyn Owen, Sandrine Silvente-Poirot, Marc Poirot

**Affiliations:** 1 Division of Preventive Oncology, National Center for Tumor Diseases, Im Neuenheimer Feld 460 and German Cancer Research Center (DKFZ), Im Neuenheimer Feld 581, Heidelberg 69120, Germany; farid_khallouki@yahoo.fr (F.K.); Robert.Owen@nct-heidelberg.de (R.W.O.); 2 Team of Endocrinology and Nutrition Physiology, Faculté des Sciences et Techniques d’Errachidia (FSTE), Université Moulay Ismail, 509, Boutalamine, Errachidia 52000, Morocco; 3 Cancer Research Center of Toulouse, Unité Mixte de Recherche (UMR) 1037 Institut National de la Santé et de la Recherche Médicale (INSERM)-University of Toulouse III, Toulouse F-31037, France

**Keywords:** cholesterol, metabolism, cholesteryl esters, tumour promoter, ChEH, cancer cell, invasiveness, colony formation

## Abstract

Bryonolic acid (BrA) is a pentacyclic triterpene present in several plants used in African traditional medicine such as *Anisophyllea dichostyla* R. Br. Here we investigated the in vitro anticancer properties of BrA. We report that BrA inhibits acyl-coA: cholesterol acyl transferase (ACAT) activity in rat liver microsomes in a concentration-dependent manner, blocking the biosynthesis of the cholesterol fatty acid ester tumour promoter. We next demonstrated that BrA inhibits ACAT in intact cancer cells with an IC_50_ of 12.6 ± 2.4 µM. BrA inhibited both clonogenicity and invasiveness of several cancer cell lines, establishing that BrA displays specific anticancer properties. BrA appears to be more potent than the other pentacyclic triterpenes, betulinic acid and ursolic acid studied under similar conditions. The inhibitory effect of BrA was reversed by exogenous addition of cholesteryl oleate, showing that ACAT inhibition is responsible for the anticancer effect of BrA. This report reveals new anticancer properties for BrA.

## 1. Introduction

**Figure 1:**
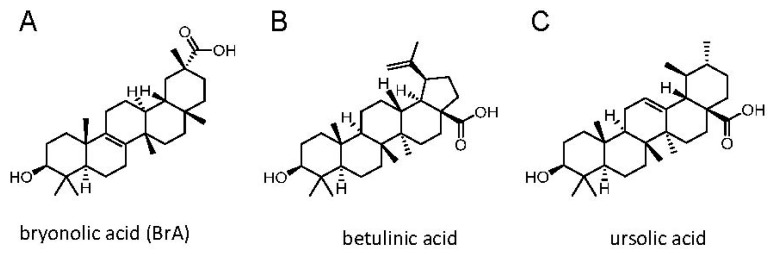
Chemical structures of the tested triterpenoid compounds.

Natural compounds are an important source of pharmacologically active molecules that have been continuously characterized for years in terms of structure and pharmacological potencies and use for drug development [1]. Among the natural molecules of plant and microorganism origin, triterpenoids occupy a central role. Triterpenoids constitute a large group of polycyclic compounds derived from squalene or 30-carbon acyclic equivalents [2]. Triterpenoids include biologically active sterols, steroids and saponins. The long extended acyclic precursors are the substrate of various enzymes, which can transform them into stereo-selective polycyclic compounds [2]. Bryonolic acid (BrA) (Figure 1A) is a triterpenoid compound detected and identified in the root of various Cucurbitaceae [3] as well as in the Coriaceae [4] and Anisophylleaceae families [5].

Pentacyclic triterpenoids have been reported to display pharmacological properties suggesting they may have utilities in the protection against several degenerative diseases including cancers [1,6,7,8,9,10]. Deregulation of cholesterol metabolism are involved in various pathologies, including cardiovascular diseases, neurodegenerative diseases [11,12], the control of the immune response [13,14], cancer [15,16,17,18] and ageing [19,20]. We recently reported the chemopreventive properties of several natural substances such as the mammalian steroidal alkaloid dendrogenin A [17,21,22,23,24], auraptene, a prenyloxycoumarin from Citrus species [25] and vitamin E including tocopherol and tocotrienol compounds [26]. We found that these compounds had capacities to inhibit different mitogenic pathways in cancer cells. These pathways include the oestrogen receptor alpha (ERα), cholesterol-5,6-epoxide hydrolase (ChEH) and acyl-co-A cholesterol acyl transferase (ACAT). The ACAT catalysed the esterification of cholesteryl esters with fatty acids and these fatty acid:cholesteryl esters were recently shown to act as tumour promoters making ACAT a new target in cancer research [13,18,25,27,28,29,30]. We report that BrA displayed anticancer properties through the inhibition of cholesterol esterification.

## 2. Materials and Methods

### 2.1. Chemicals and Reagents

Bryonolic acid (BrA) was purified and characterized exactly as described earlier [5]. Betulinic and ursolic acids were purchased from Extrasynthese (Lyon Nord, Genay, France). Acetic acid, acetonitrile, dichloromethane (DCM) and anhydrous sodium sulphate were obtained from Merck (Darmstadt, Germany). 3-[decyldimethylsilyl]-*N*-[2-(4-methylphenyl)-1-phenylethyl]-propanamide (Sah 58-035) was kindly provided by A. Suter at Novartis (Basel, Switzerland). [^3^H]17βEstradiol, [^14^C]oleyl-CoA and [^14^C] cholesterol were from Perkin Elmer (Waltham, MA, USA). The radiochemical purity of the compounds was verified by thin-layer chromatography (TLC) and was greater than 98%. Solvents were from Sigma, Fischer, Scharlau or VWR. TLC plates were from Macherey Nagel. Normal silica Sep-Pack cartridges were from Waters. All other chemicals and reagents were purchased from Sigma-Aldrich (Saint-Louis, MO, USA).

### 2.2. Assays for Cholesterol Esterification (ACAT) Inhibition

Rat liver microsomes were prepared as described previously [31]. The 105,000 g microsomal pellet was resuspended in 0.1 M phosphate buffer, pH 7.4, 1 mM EDTA and 2 mM dithiothreitol at a protein concentration of 5 mg/mL. The ACAT activity was assayed by measuring the formation of cholesteryl [^14^C]oleate from endogenous cholesterol in the microsomal fraction and exogenous [^14^C]oleyl-coA as the substrate, following the procedure described previously [31]. ACAT activity is expressed as the percentage of the activity measured in the absence of inhibitors (control assay with solvent vehicle). ACAT activity was assayed using 40 μM [^14^C]oleyl-CoA in the presence or absence of 1, 5, 10, 25 and 50 μM concentrations of the tested compounds. The concentration of compound required to inhibit ACAT by 50% (IC_50_) was calculated using Prism software, version 4.0 (GraphPad Software Inc., San Diego, CA, USA). The IC_50_ values were calculated with data from triplicate assays at each drug concentration.

### 2.3. Cell Culture

MCF-7, MDA-MB231 (MB-231) and U-87 cells were from the American Type Culture Collection (Manassas, VA, USA) and NIH-3T3-CCK2R-E151A cells (3T3-EA) were obtained as described previously [32]. MCF-7 were routinely grown in RPMI 1640 growth medium containing 5% foetal bovine serum (FBS) (Invitrogen, Carlsbad, CA, USA), 2 mM glutamine and 50 U/mL penicillin and 50 U/mL streptomycin. E151A were grown in DMEM containing 10% FBS, 2 mM glutamine and 50 U/mL of both penicillin and streptomycin. Cells were incubated at 37 °C in a humidified 5% CO_2_/air atmosphere.

### 2.4. Assay for ACAT Activity in Intact Cells

MCF-7, MB-231, U-87 and 3T3-EA cells were plated on six-well plates (40,000 cells/well). ACAT activity in intact cells was measured as described previously [25]. Cells were preincubated for 15 min. with solvent vehicle or increasing concentrations of BrA ranging from 1 to 200 μM in complete medium. [^14^C]Cholesterol (0.2 μCi/well) was added and the cells were incubated for 24 h. At the end of the incubation, intracellular and secreted lipids in the supernatant were extracted and then separated by TLC as described previously [30]. Free and esterified cholesterol were identified using purified ^14^C commercial standards and the radioactivity of each individual lipid was quantified using a phosphor screen (Storm; GE Healthcare, Chicago, IL, USA). ACAT activity is expressed as the percentage of the ACAT activity measured in the absence of inhibitors (cells treated with solvent vehicle).

### 2.5. Oestrogen Receptor Binding Assay

Competition binding to ERα and ERβ was measured exactly as described previously [26].

### 2.6. Cholesterol Epoxide Hydrolase (ChEH) Assays

Inhibition of ChEH activity was measured as described previously on a whole-cell assay using MCF-7 cells [33]. [^14^C]α-CE (10 Ci/mol) was synthesized as described previously [34]. Cells were treated for 24 h with 0.6 μM [^14^C]α-CE. The final assay volume was 150 μL containing 130 μL of buffer (50 mM Tris, pH 7.4 and 150 mM KCl), 10 μL of microsomal proteins (15 mg/mL) and 10 μL of acetonitrile (6.7%) containing the test compound/drug and the labelled α-CE. 

The mixtures were incubated over a period of 0 to 30 min. The tubes were placed in ice cold distilled water to stop the reaction and the addition of 1.5 mL chloroform/methanol (2:1) and reaction buffer (500 mL) followed. After vortexing, the lower phase was aspirated and the residual aqueous phase was extracted with chloroform (1.5 mL). The organic extracts were combined and dried under a gentle stream of nitrogen, followed by suspension in ethanol (60 µL). Over 90% of the radioactivity was present in the organic extracts.

Samples were applied to TLC plates that had been heated previously for 1 h at 100 °C and were developed using ethyl acetate. The regions corresponding to authentic CE and cholestane-3β,5α,6β-triol standards were visualized by iodine vapour. Radioactive metabolites were visualized using a Storm apparatus (GE Healthcare) and quantified by densitometry with the software ImageQuant version 5.2 (GE Healthcare). Cell culture: 3T3-EA was obtained as previously described [32] and was grown in DMEM medium containing 10% foetal bovine serum (Invitrogen, Carlsbad, CA, USA), 2 mM glutamine, 50 units/mL of both penicillin and streptomycin. Cells were incubated at 37 °C in a humidified 5% CO_2_/air atmosphere at 37 °C. 

### 2.7. Measurement of the Effect of Compounds on Cholesterol Esterification

Cells were plated on 6-well plates. The cells were preincubated for 15 min with solvent vehicle, Sah 058-035 (10 µM), BrA (50 μM), ursolic acid (50 µM) and betulinic acid (50 μM) in complete medium. [^14^C]cholesterol (0.2 μCi/well) was added and cells were incubated for 24 h. At the end of the incubation, intracellular lipids were extracted as described in [30] and then separated by TLC as described previously [11]. Free and esterified cholesterol were identified using purified [^14^C] standards and the radioactivity associated with each individual lipid was quantified using a Phosphoscreen (Storm; Molecular Dynamics). ACAT activity is expressed as the percentage of the ACAT activity measured in the absence of inhibitors (cells treated with solvent vehicle). 

### 2.8. Clonogenic Assay

Following trypsinization of the cells, they were plated in tissue culture plates (60 mm) at a density of 500–1000 per plate. After adherence for 24 h, the drugs were added at the chosen final concentrations from freshly prepared stock solutions. The plates were washed twice with serum-free medium after 24 to 72 h incubation. Fresh medium was added and the plates were incubated at 37 °C until the colonies became visible. After incubation, the plates were washed with PBS and stained using Coomassie brilliant blue. The stained colonies were counted and are reported as the percentage of control cells (ethanol treated (0.01% (*v*/*v*)).

### 2.9. Cell Invasion Assays

Cells were added to six-well plates (40,000 cells/well) containing Dulbecco's Modified Eagle Medium (DMEM) and 10% FCS. Following incubation for 24 h, the cells were treated for a further 24 h in the presence of the test compounds, or else vehicle in DMEM and 2% Fetal Calf Serum (FCS). The cells were harvested and counted. Cells (20,000) were placed in serum-free DMEM on the surface of Nunc filters (8 mm diameter, 8 mm pore size; Nalgen Nunc International, Rochester, NY, USA). The filters were coated with growth factor-reduced Matrigel (250 mg/mL Matrigel; BD Biosciences, San Jose, CA, USA) in the presence of the test compounds or else vehicle. The base of the filter was filled with 10% FCS/DMEM. Following incubation for 48 h at 37 °C, cells which had invaded the Matrigel and attached themselves to the base of the filter were fixed, stained with Giemsa stain and counted microscopically.

### 2.10. Statistical Analysis

Values are the mean ± S.E.M. of three independent experiments, each carried out in duplicate. Statistical analysis was carried out using the Student’s *t*-test for unpaired variables. *, **, *** in the Figure refer to *p*-values of <0.05, <0.001 and <0.0001 respectively, compared with control cells that were treated with solvent vehicle alone.

## 3. Results and Discussion

Bryonolic acid (D-C friedoolean-8-en-3β-ol-29 oic acid) belongs to the family of the D:C fridooleane and this structural class of triterpene has interesting stereochemical features and biological activities [7]. We tested here if BrA could affect cancer cell proliferation and invasiveness. Cholesteryl esters have been recently identified as tumour promoters by showing they stimulated cancer cells clonogenicity and invasiveness and the inhibition of the cholesteryl esterification of fatty acids (ACAT) has been shown to represent a promising target for cancer management [18,29,30,35,36,37]. Several pentacyclic triterpenoids including betulinic acid and ursolic acid were reported to inhibit ACAT [38,39,40] suggesting that other pentacyclic triterpenoids could be ACAT inhibitors. We first investigated the effect of BrA on ACAT activity from rat liver microsomes and established that it inhibited cholesterol esterification in a concentration-dependent manner (Table 1). 

The measured IC_50_ was 12.6 ± 2.4 µM. Betulinic acid and ursolic acid inhibited ACAT with an IC_50_ of 18.5 ± 2.1 µM and 71.4 ± 5.1 µM respectively (Table 1). 

To determine whether this inhibition of ACAT measured in vitro was equivalent in cancer cells, we measured ACAT inhibition in whole cell assays as previously reported [25,30]. We found that BrA potently inhibits ACAT on different cancer cell lines and in a genetically engineered cell line in which cholesteryl esters were shown to be responsible of their tumorigenicity (Table 2). BrA displayed an efficacy on whole cells, in the same range as in the in vitro assay while a decrease of efficacy and a loss of ACAT inhibition was observed with betulinic acid and ursolic acid respectively (Table 2).

We next investigated the putative impact of BrA on other targets involved in the chemoprevention of breast cancers such as ERα [41] and cholesterol-5,6-epoxide hydrolase (ChEH) [15,21,42] and found that BrA does not modulate ER and does not inhibit ChEH (Table 3).

**Figure 2:**
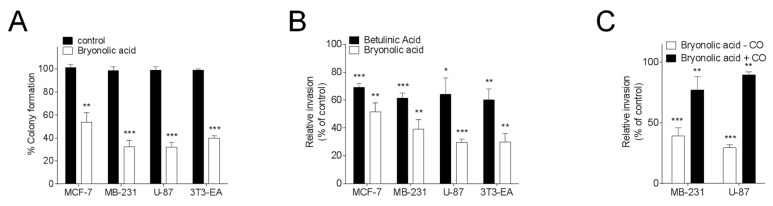
(**A**) Effect of Bryonolic acid on colony formation. Cells were treated with 25 µM BrA and the number of colonies was measured compared with solvent vehicle-treated cells (taken to be 100%); (**B**) after 24 h of pre-treatment with either the solvent vehicle or 25 μM betulinic acid or BrA, cell invasion was assayed using Matrigel-coated filters as described under Materials and Methods. After 48 h, cells on the lower surface of the filters were stained and counted under a phase-contrast microscope; (**C**) Effect of cholesteryl oleate on the inhibition of MB-231 and U87 cell invasiveness by BrA. Cells were pre-treated for 24 h with cholesteryl oleate (4 µg/mL) and layered on the top of Matrigel-coated filters in serum-free medium for 48 h (+CO) or cells were treated similarly with the solvent vehicle (−CO). After 48 h, cells on the lower surface of the filters were stained and counted. Values are expressed relative to that of cells treated with the solvent vehicle (−CO) and are the mean ± SEM of three separate experiments. Values are expressed relative to those of cells treated with the solvent vehicle (control) and are the mean ± S.E.M. of three to six separate experiments performed in triplicate. (* *p* < 0.05, ** *p* < 0.01, *** *p* < 0.001).

Because cholesteryl esters are tumour promoters, we next investigated the effect of BrA on cell clonogenicity and invasiveness. We found that 25 µM BrA inhibited cancer cell colony formation by more than 50% in the four different tested cell lines (Figure 2A). 

Tests on invasiveness were also positive and BrA showed a better efficacy than betulinic acid in this effect. The inhibition of invasiveness measured for BrA paralleled the ACAT inhibition measured on the whole cell assays. The inhibition of invasiveness induced by BrA was observable in the four cell lines (Figure 2B). The less potent effect being on the breast cancer cell line MCF-7 in which cholesterol esterification is weaker than in other tested cell lines [25,30,43]. Next, we found that the addition of cholesteryl oleate to cells treated with BrA blocked the inhibition of cell invasiveness triggered by BrA (Figure 2C). Taken together these data shows that BrA is more potent that betulinic acid and ursolic acid in the inhibition of cell invasiveness showing that BrA in vitro blocks the most poisonous parameters in cancer development, which are tumour invasiveness and colony formation.

BrA is a pentacyclic triterpene present in several plants. Although some pharmacological properties were proposed for BrA, little was known on its putative impact on cancer cells, except on cytotoxicity [8]. We thus investigated its impact on cholesterol esterification on liver extracts and on whole cell assays and established that BrA was a potent ACAT inhibitor. BrA was less potent than the prototypical ACAT inhibitor Sah 58-035 but more potent than two other pentacyclic terpenoids betulinic acid and ursolic acid that are well studied for their pharmacological properties [7,44,45]. Although ACAT inhibition was earlier used to screen compounds with steroidal backbones for putative antiatheromatous properties [31], recent data from the literature showed that cholesteryl esters of fatty acids displayed tumour promoter properties and that ACAT inhibition in cancer cells blocked cancer cell invasiveness and clonogenicity and activate the lymphocyte T CD8+ antitumor activity [13,18,27,29,30,35,36]. We report here for the first time that BrA displays ACAT inhibition and inhibits cancer cell clonogenicity and invasiveness. This effect was observable on the ERα positive breast cancer cell line MCF-7, on the triple negative breast cancer cell line MDA-MB-231, on the glioblastoma U-87 and on the transgenic tumorigenous cell line 3T3-EA. This suggests that BrA could display anticancer properties on cancer cells of different tissue origin, opening up a broad range of putative anticancer applications for BrA. Its impact on in vivo tumour cancer in curative and chemopreventive settings deserves further investigation. 

## Figures and Tables

**Table 1:** Effect bryonolic acid, betulinic acid and ursolic acid on cholesterol esterification (ACAT activity). ACAT activity was measured on in rat liver microsomes. Rat liver microsomes (60 μg of protein) were incubated with 10 different concentrations of each drug and 40 μM [^14^C]oleyl-CoA. Cholesteryl esters were quantified as described under the “Materials and Methods” section. ACAT activity is expressed as the percentage of the ACAT activity measured in the absence of inhibitors (control with solvent vehicle).

Compounds	IC_50_ in µM
sah 58-035	0.65 ± 0.22
bryonolic acid	12.6 ± 2.4
betulinic acid	18.5 ± 2.1
ursolic acid	71.4 ± 5.1

**Table 2:** Inhibition of ACAT activity in intact MCF-7, MB231, U-87 and 3T3-EA cells. Cells were treated as described in the legend of Table 1 for 24 h. Free and esterified cholesterol were quantified as described in the “Materials and Methods” section. ACAT activity is expressed as the percentage of the ACAT activity measured in the absence of inhibitors (control with solvent vehicle). IC_50_ values were determined using the iterative curve fitting program GraphPad Prism (version 6.0). Values are the average of three experiments ± S.E.M., each carried out in duplicate. n.m.: not measurable.

	MCF-7	MB-231	U-87	3T3-EA
**Compounds**	**IC_50_ in µM**			
sah 58-035	5.1 ± 0.5	9.1 ± 1.4	9.2 ± 2.2	7.4 ± 1.8
bryonolic acid	22.5 ± 3.7	29.5 ± 5.5	17.5 ± 4.8	19.4 ± 7.6
betulinic acid	53.7± 4.2	58.3 ± 8.1	61.4 ± 9.4	60.2 ± 8.0
ursolic acid	n.m.	n.m.	n.m.	n.m

**Table 3:** Competition assays on ERs and measure of ChEH inhibition. Binding of radiolabelled 17β-estradiol (E2) to the ERα and ERβ were measured at different concentrations of compounds as described under “Materials and Methods.” For the ChEH inhibition tests, 150 μg of rat liver microsomal proteins and 10 and 20 μM concentrations of [^14^C]α-CE with increasing concentrations of compounds ranging from 0.01 to 1000 μM were used under the conditions described under Materials and Methods. Values are the mean ± S.E.M. from three independent experiments. n.m., no measurable inhibition of binding.

Compounds	ERα	ERβ	ChEH
**IC_50_ in nM**			
E2	1.2 ± 0.5	1.7 ± 0.7	n.m.
tamoxifen	45.7 ± 5.1	58.4 ± 7.1	122.6
bryonolic acid	n.m.	n.m.	n.m.
betulinic acid	n.m.	n.m.	n.m.
ursolic acid	n.m.	n.m.	n.m.

## Abbreviations

BrA	Bryonolic acid
Sah 058-035	3-[decyldimethylsilyl]-*N*-[2-(4-methylphenyl)-1-phenylethyl]-propanamide
ACAT	Acyl-CoA:cholesterol Acyl Transferase
